# High-Precision Oxygen-Isotope Analysis of Iron (Oxyhydr)oxides Using High-Temperature Conversion Isotope Ratio Mass Spectrometry

**DOI:** 10.1021/acs.analchem.4c04676

**Published:** 2025-03-28

**Authors:** Nir Galili, Anna Somlyay, Giorgia Aquila, Reto Wijker, Philip Gautschi, Lukas Wacker, Jordon D. Hemingway

**Affiliations:** † Geological Institute, Department of Earth Sciences, ETH Zurich, 8092 Zurich, Switzerland; ‡ Laboratory of Ion Beam Physics, Department of Physics, ETH Zurich, 8093 Zurich, Switzerland

## Abstract

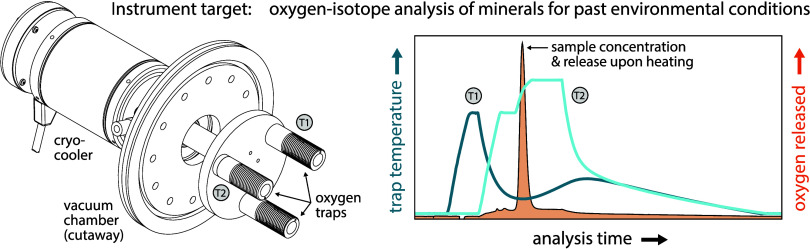

We introduce a novel high-precision method for oxygen-isotope analysis of iron (oxyhydr)oxides using high-temperature conversion isotope ratio mass spectrometry (HTC-IRMS). In this approach, a finely ground mixture of iron (oxyhydr)oxide and graphite is heated at 1450 °C in a helium flow environment, converting oxygen to CO gas with nearly 100% yield. Continuous-flow IRMS analysis of the liberated CO yields a precision of ±0.15‰ (1σ, *n* = 28) and shows excellent agreement with (and improved precision over) traditional fluorination methods. This practical and safe technique expands access to oxygen-isotope measurements of iron oxides, thereby enhancing their utility in Earth and environmental sciences.

## Introduction

Iron (oxyhydr)oxide minerals are ubiquitous in several environmental contexts; their abundance, mineralogy, and isotopic compositions offer critical insight into various processes and past climatic conditions.^1−3^ Among the oxidized, iron(III)-containing (oxyhydr)oxides, goethite (α-FeOOH) and hematite (α-Fe_2_O_3_) are the most prevalent on Earth; they form under diverse low-temperature, surface environments.^3^ In addition to their abundance and mineralogy, the stable isotopic compositions of these minerals represent an essential tracer for understanding the temperature, parent-water composition, and environmental pH under which they formed. Of particular interest is their oxygen-isotopic composition (^18^O/^16^O, reported as δ^18^O), as this has been shown to represent a quantitative proxy for past hydrologic conditions that is robust to postformation alteration.^1,2^ For example, oxygen-isotope measurements of goethite and hematite have been used to reconstruct hydrologic variability on time scales ranging from millennia to billions of years.^1,2^ This measurement therefore forms an integral component of paleohydrology and paleoclimate research.

Despite this utility, iron (oxyhydr)oxide δ^18^O analysis has historically relied heavily on specialized fluorination techniques that are available only in a small number of laboratories worldwide. Early methodologies involved the use of nickel (Ni) reaction tubes (so-called “bombs”), in which samples are heated at 500–650 °C in the presence of halogen gases such as fluorine (F_2_) or bromine pentafluoride (BrF_5_).^4,5^ These methods effectively liberate oxygen from the mineral matrix but require labor-intensive and complex handling due to the reactive and hazardous nature of the gases involved. Beyond safety and handling issues, obtaining quantitative oxygen yields using Ni-bomb techniques has proved challenging,^6,7^ thus necessitating extensive testing and cross-validation to ensure that isotopic results are robust and that fractionation during sample handling is minimized or well-constrained.^8^

To alleviate some handling and throughput issues, laser-fluorination-isotope ratio mass spectrometry (LF-IRMS) methods—in which high-powered lasers are used to “spot heat” a sample to liberate oxygen—were subsequently developed.^1,9,10^ These techniques are often employed “in line” with the mass spectrometer (i.e., they do not require breaking a vacuum seal and exposing surfaces to atmosphere), thus improving precision.^1,9^ Furthermore, by requiring less sample material relative to Ni bombs, LF-IRMS allows one to capture natural heterogeneity at smaller spatial scales. This method has therefore grown to represent the dominant means by which iron (oxyhydr)oxide isotopic compositions are analyzed today.

Nevertheless, like for Ni bombs, LF-IRMS employs highly oxidizing gases such as F_2_, BrF_5_, or chlorine trifluoride (ClF_3_).^1,9^ Thus, despite improvements in the sample size and precision, this technique still requires strict safety protocols and extensive preparation and handling time. Furthermore, several authors have reported less-than-quantitative yields for several minerals using LF-IRMS, likely due to a combination of cold spots, sample sputtering, user variability in the laser handling technique, and the formation of competing side products (i.e., M_*x*_O_*y*_F_*z*_, where M is any third element and *x*,*y*,*z* ≥ 1).^7,8,11^ LF-IRMS methods therefore still require extensive testing and cross-validation to quantify and minimize the analytical isotope fractionation that results from incomplete yields. In total, the safety, handling, and throughput limitations of both Ni-bomb and LF-IRMS techniques to date have limited the practicality for oxygen-isotopic studies of iron (oxyhydr)oxides.

As an alternative, here, we introduce a new method to measure δ^18^O values of goethite and hematite using high-temperature conversion isotope ratio mass spectrometry (HTC-IRMS). In HTC-IRMS, oxygen is liberated as carbon monoxide (CO) from a finely ground mixture of iron (oxyhydr)oxide and graphite in a glassy carbon furnace at 1450 °C under a helium (He) flow. Liberated CO is then isolated and cryofocused in a custom-built trapping interface before being analyzed by traditional continuous-flow isotope ratio mass spectrometry (IRMS).^7,8^ As demonstrated below, this technique allows for fully automated, rapid sample processing; results in quantitative oxygen yields; and maintains precision better than those achieved by LF-IRMS. Thus, relative to traditional fluorination techniques, our approach significantly reduces safety risks and increases sample throughput and data quality. By offering a more efficient and practical solution for iron (oxyhydr)oxide δ^18^O analysis, this method aims to advance research into the geochemical processes shaping our rusting planet.

## Experimental Section

### Isotope Notation

Oxygen-isotope ratios are represented relative to the international standard Vienna Standard Mean Ocean Water (VSMOW) and are often expressed in traditional “delta” notation as
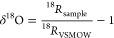
1where ^18^R denotes the ^18^O/^16^O ratio. Typically, δ^18^O values are reported in units of “permil” (‰) by multiplying eq 1 by 1000. In addition, since traditional δ^18^O values are nonlinear over large isotopic ranges, we linearize isotopic relationships by transforming δ^18^O values to a logarithmic scale. Specifically, we calculate “delta-prime” values as
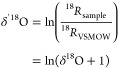
2which are similarly reported in units of permil by multiplying eq 2 by 1000. Throughout this study, we use δ′^18^O values when calibrating our reference gas and determining IRMS linearity (i.e., since reference materials used here span a >150‰ range), but we report all iron (oxyhydr)oxide results as traditional δ^18^O values (i.e., to compare with published values and because these only span a ∼5‰ range).

### Sample Synthesis

To obtain large quantities of mineralogically pure materials for method development and calibration, we synthesized both goethite and hematite under a range of environmental conditions. Additionally, δ^18^O values of all synthesized materials used in this study have been measured previously using traditional laser fluorination with continuous-flow IRMS,^1^ thus providing a direct means to compare methodologies. Synthesis procedures are briefly described in the Supporting Information (additional details in ref (1)). All synthesized FeOOH and Fe_2_O_3_ samples exhibit a uniform grain size of less than 20 μm, minimizing the variability in reaction kinetics during the pyrolysis process and potential isotopic fractionation.

### HTC-IRMS Instrumental Design

We describe the HTC-IRMS instrument, including a custom-built trapping interface for cryofocusing carbon monoxide (CO) gas and separating isobarically interfering contaminant gases (e.g., N_2_). We sequentially detail the below: (i) HTC elemental analyzer, (ii) trapping interface, and (iii) IRMS setup.

#### HTC Elemental Analyzer

The elemental analyzer converts iron (oxyhydr)oxide-bound oxygen to CO gas in the presence of elemental carbon (C(s)) at elevated temperatures in a reducing environment. For our minerals of interest, Ellingham diagrams indicate these reactions are thermodynamically favorable (i.e., Δ_*r*_*G* ≤ 0 kJ mol^–1^) above ∼750 °C;^12,13^ nevertheless, here, we employ a furnace temperature of 1450 °C to accelerate reaction kinetics as much as possible within practical furnace limits.^14−16^ At these temperatures, goethite and hematite decomposition is expected to follow the reactions
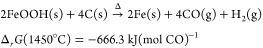
3and
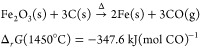
4respectively, where Δ indicates the addition of heat.

**Figure 1:**
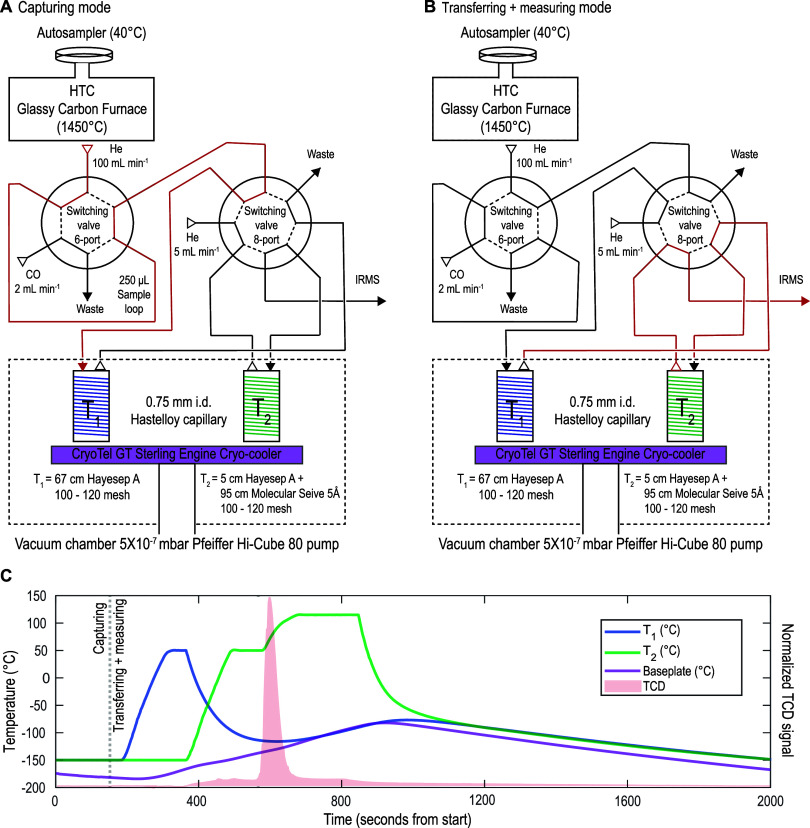
HTC-IRMS schematic diagram. The system contains two primary operational modes, (A) CO capturing and (B) CO transferring + measuring, and is toggled between the two using an 8-port switching valve. In capturing mode, CO gas is cryogenically frozen on *T*_1_ (blue coil) at −150 °C; CO is either generated in the elemental analyzer furnace at 1450 °C (samples, blanks, mineral standards) or is introduced via a sample loop and 6-port switching valve (reference gas standard) immediately downstream of the elemental analyzer. In both cases, CO is carried to *T*_1_ in 100 mL min^–1^ of He carrier gas (red flow path in panel (A)). In transferring + measuring mode, CO gas is released from *T*_1_ at 50 °C and is refrozen on *T*_2_ at −150 °C (green coil) for cryofocusing before being released to the IRMS via two-step heating at 50 and 115 °C to achieve chromatographic N_2_ and CO separation. In this mode, CO gas is carried to *T*_2_ and subsequently to the IRMS for isotopic analysis at a reduced He carrier gas flow rate of 5 mL min^–1^ (red flow path in (B)). Both traps are cryogenically cooled via a copper baseplate mounted to a Sterling engine cryocooler (purple plate) and heated via direct electrical resistance; the entire apparatus is housed within a vacuum chamber to minimize heat transfer (black dotted box). (C) Temperature profiles and TCD signals for a typical analysis. Colors correspond to system components as introduced in panels (A) and (B) (blue = *T*_1_, green = *T*_2_, purple = baseplate, red = TCD signal indicating CO release), whereas the vertical gray dotted line indicates timing of switch from capturing to transferring + heating mode.

As seen on the left-hand side of eqs 3 and 4, it is crucial that C(s) is provided in excess to promote the forward reactions and quantitatively liberate the CO gas. To achieve this, all HTC reactions in this study were conducted using a Thermo Scientific (Bremen, Germany) EA IsoLink OH instrument equipped with a Costech Analytical (Valencia, CA, USA) ZeroBlank autosampler flushed with reference He gas (Figure 1A,B). The IsoLink OH furnace features a glassy carbon pyrolysis tube (365 mm length; 12 mm o.d.; 7 mm i.d.; Säntis Analytical AG, Teufen, Switzerland) housed inside a larger ceramic tube that serves as a purge housing (450 mm length; 17 mm o.d.; 13 mm i.d.; Säntis Analytical AG); He carrier gas is introduced from below to minimize precipitation of metals and reduce the CO blank (so-called “bottom-feed adapter”). Before each analytical session, the glassy carbon tube is packed first with 5 mm quartz and silver wool, then with ∼100 mm glassy carbon chips, and finally with a graphite crucible that rests in the furnace “hot spot”.

Upon exiting the furnace, the produced analyte gas is quantified using a thermal conductivity detector (TCD) situated downstream of the furnace outflow. HTC instruments typically operate with a packed gas chromatographic column between the furnace and TCD, which separates the CO from the contaminant gases; however, we bypass this column in favor of our custom-built trapping interface (see below). Finally, the analyte gas passes through a packed glass trap (110 mm; 12 mm o.d.) containing scaffolded NaOH on silica (CO_2_ absorbing agent; Thermo Scientific) and magnesium perchlorate (Mg(ClO_4_)_2_; dehydrating agent; Thermo Scientific) in series before being transferred to our custom trapping interface via a 1/16” o.d., 0.75 mm i.d. electropolished Ni capillary (VICI Valco Instruments, Houston, TX, USA).

#### Trapping Interface

After exiting the HTC elemental analyzer, analyte gas is purified and cryofocused by using our custom-built trapping interface (Figure 1A,B). This interface, based on similar instrumental designs,^17−21^ features two packed adsorbent capillary traps whose temperature can be independently manipulated between ∼−200 °C and ∼150 °C via a combination of electrical resistance heating and contact with a cryocooled copper baseplate. Briefly, CO is cryogenically frozen onto the first trap (*T*_1_), transferred to the second trap (*T*_2_) where it is chromatographically separated from contaminant gases and quantified, and sent to the IRMS using continuous flow. Analyte gas is either generated from a sample in the HTC furnace (see above) or introduced as a pulse of CO reference gas via a 250 μL sample loop just downstream of the elemental analyzer (Figure 1A,B). The latter serves as a means of monitoring any isotope fractionation introduced by our trapping interface system.

Specific to our instrument, capillary traps (100 cm cooled/heated trap length; 1/16 in. o.d.; 0.75 mm i.d.) are constructed from Hastelloy C alloy (VICI Valco Instruments) to maximize electrical resistance and minimize reactivity with corrosive gases. Because the purpose of *T*_1_ is to cryogenically freeze CO gas from the HTC elemental analyzer outflow stream, it is filled with 67 cm of packed HayeSep A porous polymer adsorbant (100/120 mesh; Hayes Separation Inc., Bandera, TX, USA); this material has been shown to effectively sorb CO and other permanent gases (e.g., refs (17−22) and HayeSep white papers). Because the purpose of *T*_2_ is to chromatographically separate CO from contaminant gases, particularly N_2_, at lower flow rates, it is filled with 5 cm of packed HayeSep A for cryofocusing followed by 95 cm of a 5 Å molecular sieve (100/120 mesh; Ohio Valley Specialty Company, Marietta, OH, USA) to achieve separation. Both traps are plugged with stainless steel mesh to hold the adsorbent in place and were heated at ∼150 °C for ∼12 h under 5 bar N_2_ flow prior to winding to ensure optimum bed packing. Stained thick-section microscopy images on a packed column cross section indicate that a final porosity of ϕ ∼ 11% was achieved (Figure S1).

To prevent overheating of portions of the capillary that are not in thermal contact with the baseplate, the final 25 cm of both sides of both traps (i.e., leading to a total capillary length of 150 cm) are coated in 100 mm copper (Galvotec GmbH, Schöfflisdorf, Switzerland). Copper-coated sections are surrounded by 1/8” o.d. PEEK tubing, and one end is inserted into a PEEK and the other into a Hastelloy C zero dead volume union (VICI Valco Instruments); this design precludes transfer of electricity to the rest of the trapping system. Capillary traps are heated via direct pulses of 12 V, 16A DC power (Dimension CP10.121, Puls Power, Munich, Germany) that are controlled by an 8-channel PID temperature controller (TM4-N2SB/E, Autonics Corporation, Seoul, Republic of Korea), with temperature monitored by a pt100 resistance temperature detector (model 1PT100 KN1515CLA; Omega Engineering Ltd., Manchester, England, UK).

Cryogenic temperatures are achieved using a CryoTel GT Stirling Engine cryocooler (nominal lift of 16 W at −196 °C; Sunpower Inc., Athens, OH, USA) attached to a copper baseplate (10 cm diameter, 1 cm thickness) that has been polished to ≤0.25 μm roughness to maximize the contact surface area and thus improve thermal conductivity.^21^ Heat generated by the cryocooler is rejected into a ∼1000 mL min^–1^ stream of chilled water that does not exceed 20 °C (flow rate and temperature monitored by model PF3W504S-NO3-1T-R flow sensor; SMC Corporation, Tokyo, Japan). Capillary traps are attached to the copper baseplate by coiling (17 turns) around custom-machined threaded aluminum standoffs (20 mm diameter; 55 mm height) that are coated in a 40-mm-thick anodized oxide surface (Altefco AG, Blaterswil, Switzerland) to prevent electrical conductivity between capillaries and the baseplate; standoff bases are similarly polished to ≤0.25 mm roughness to maximize the contact surface area.^17,21^ Thermal contact is further improved by applying a thin layer of Dowsil 340 thermoconductive paste (Dow Corning Corp., Midland, MI, USA) between capillary traps and standoffs. To minimize heat loss to the surrounding atmosphere, the entire baseplate and trap apparatus is housed within a custom-machined cylindrical vacuum chamber (150 mm inner diameter, 140 mm depth) featuring a borosilicate glass viewing window. High vacuum (≤10^–6^ mbar) is achieved using a HiCube 80 Eco turbo pump and is monitored by model PKR 360 Compact Full Range vacuum gauge (Pfeiffer Vacuum GmbH, Aßlar, Germany).

All carrier gas used in this study is 6.0-grade He that is further purified using a heated helium purifier (model HP2-220; VICI Valco Instruments) to remove trace permanent gases, halogenated gases, and light hydrocarbons. He and CO flow rates are controlled using calibrated mass flow controllers (model MFC2022-AI/BE-S2; Axetris AG, Kägiswil, Switzerland). Gas flows are manipulated via two switching valves with 1/16 in. fittings: a VICI Valco 6-position selector valve with an electronic actuator (model EUTA-CSTF6MWENIPH) and a VICI Valco 6-port switching valve with a pneumatic actuator (model AC6UWEPI). Although the former is technically a selector valve, all methods used in this study toggle between only two states; this valve is thus represented instead as an 8-port switching valve in Figure 1 for simplicity. Both valves are constructed of Ni metal bodies to reduce reactivity with corrosive gases and are internally purged with He to minimize the inward diffusion of air.

To monitor carrier gas pressure, a pressure sensor (model PAA-4LD, Keller Druckmesstechnik AG, Winterthur, Switzerland) is installed upstream of *T*_1_. Pressures were consistently held at ∼3 bar throughout all analyses by the use of custom capillary crimps located on the HTC and trapping interface outflow capillaries. Similarly, to monitor and quantify chromatographically separated gases, a custom-built TCD based on MEMS technology (model TC-1326-A, SGX Sensortech, Neuchâtel, Switzerland) is installed downstream of *T*_2_. All capillaries within the trapping interface—with the exception of Hastelloy C traps—are constructed of electropolished Ni (1/16 in. o.d., 0.75 mm i.d.; VICI Valco Instruments).

The entire interface system is controlled by a custom-built electronics control board (i.e., for power distribution and I2C and UART signal processing) that communicates via a Raspberry Pi Pico microcontroller (Raspberry Pi Foundation, Cambridge, England, UK). All electronics are powered by either 24 or 48 V DC power (model EDR-120-24 or NDR-480-48, respectively; Mean Well Enterprises Co., Ltd., Taipei, Taiwan). Finally, fully automated sample processing and analysis is achieved using custom-built Micropython drivers and a user interface that communicates with Thermo Scientific’s Isodat IRMS software.

#### IRMS

Upon exiting the trapping interface, analyte gas is transferred to a Thermo Scientific Conflo IV continuous-flow gas interface and finally to a Thermo Scientific Delta V Plus IRMS with a three-cup collector assembly (i.e., *m*/*z* 28, 29, 30). Source focus settings are optimized prior to each analytical session, and all isotopic compositions are referenced to 6000 mV pulses of CO reference gas introduced at the beginning and end of each sample.

#### Session Settings

All analytical sessions in this study were performed under the following conditions: autosampler temperature of 40 °C (heated via a custom-built hot plate controlled by the same PID controller as capillary traps), HTC furnace temperature of 1450 °C, total HTC He flow rate of 100 mL min^–1^ (30 mL min^–1^ reference gas introduced via the flushed autosampler; 70 mL min^–1^ carrier gas introduced via the bottom-feed adapter). Upon loading samples and reaching set-point temperatures, the entire system is flushed for ∼12–24 h to achieve a stable baseline TCD signal prior to analysis. For each sample, blank, or reference gas pulse, the trapping interface method is as follows: trap HTC outflow on *T*_1_ for 1 min at −150 °C, transfer to *T*_2_ by heating *T*_1_ to +50 °C for 3 min, release N_2_ and other contaminant gases by heating *T*_2_ at +50 °C for 3 min, release CO by heating *T*_2_ to +115 °C for 4.5 min, return to starting temperatures (Figure 1C). All trapping interface steps are carried out in a He flow rate of 5 mL min^–1^, and samples are typically analyzed with 89% He dilution in Conflo IV.

### Analysis Workflow

#### Sample Preparation

All goethite and hematite samples are predried under N_2_ then mixed with N_2_-dried graphite (Sigma-Aldrich, St. Louis, MO, USA; prefurnaced at 500 °C for 5 h to reduce contamination) in a 1:1 mass ratio using an agate mortar and pestle. The mixture is ground finely until homogeneous in color and texture. Typically, ∼600 μg of this mixture is then weighed using a microbalance (model XPR2; Mettler Toledo, Columbus, OH, USA) into tin capsules (Säntis Analytical AG) that have been prewashed with acetone and doubly distilled water (DDW, 18.2 Mω·cm^–1^) and dried under N_2_. Capsules are not immediately sealed but rather undergo five N_2_ flushing and vacuum cycles and are left to degas for 24 h in a dry, N_2_-filled anaerobic chamber (Coy Laboratory Products, Grass Lake, MI, USA). After being degassed, capsules are sealed within the anaerobic chamber and placed in an N_2_-filled desiccator before being loaded into the autosampler. This handling procedure aims to minimize air exposure to prevent moisture and oxygen sorption. All samples are analyzed at least in triplicate. Additionally, blanks consisting of the same mass of graphite in the same tin capsules are added at the beginning, middle, and end of all analytical sessions for blank correction. Tests using both tin and silver capsules produced similar oxygen–isotope results, and the near-100% oxygen yields indicate that any capsule flash reactions are negligible; future work should further investigate potential capsule effects.

#### Oxygen Yield Determination

For each analytical session, we additionally include benzoic acid (C_6_H_5_COOH; sample IAEA-602; 26.2 wt % O) at varying masses (typically 50–1000 μg) that bracket the expected amount of CO generated by our iron (oxyhydr)oxide samples based on ideal mineral formulas. Because the TCD peak area in our trapping interface (e.g., Figure 1C) varies linearly with the quantity of CO gas trapped, we use the mass vs area relationship generated by our benzoic acid dilution series to quantify sample yields under the assumption that benzoic acid releases all of its oxygen at 1450 °C. Yields are reported as the ratio of oxygen trapped as CO to the total amount available as determined by the loaded iron (oxyhydr)oxide sample mass and assuming ideal mineral formulas.

#### Oxygen-Isotope Calibration

To constrain CO reference gas isotopic composition and HTC-IRMS linearity, we directly analyzed several liquid water international reference materials (i.e., SLAP2, GRESP, VSMOW, and IAEA-607 from the International Atomic Energy Agency; USGS-64444 and USGS-50 from the United States Geological Survey).^23−27^ Liquid samples underwent the exact same HTC and trapping procedure as described above except that the ZeroBlank solid autosampler was replaced with a Liquid Handling Kit with a high-temperature septum (Thermo Scientific); the entire HTC He flow was thus introduced via the bottom-feed adapter. Samples (≈1.8 μL) were then injected manually at least in triplicate. To correct for instrumental scale compression, liquid water standards covering a broad range in accepted δ′^18^O values (approximately 150‰) were analyzed using the same HTC and trapping procedures as for solid samples (with the autosampler replaced by a liquid handling kit). Measured values display a linear response with slope near unity over this analytical window (measured vs accepted δ′^18^O slope = 1.031 ± 0.003, *n* = 6, Figure S2, Table S1). Bracketing reference gas pulses (i.e., that underwent the exact same analytical procedure as solid samples) were then used to capture any transient fractionation effects due to specific instrumental fractionation. Calibration against IAEA-602 benzoic acid reference material further verifies that the corrected values are accurately transferred to the SMOW/SLAP scale. All iron (oxyhydr)oxide mineral results are reported after correcting to this calibration scheme.

## Results

### Oxygen Yields

**Figure 2:**
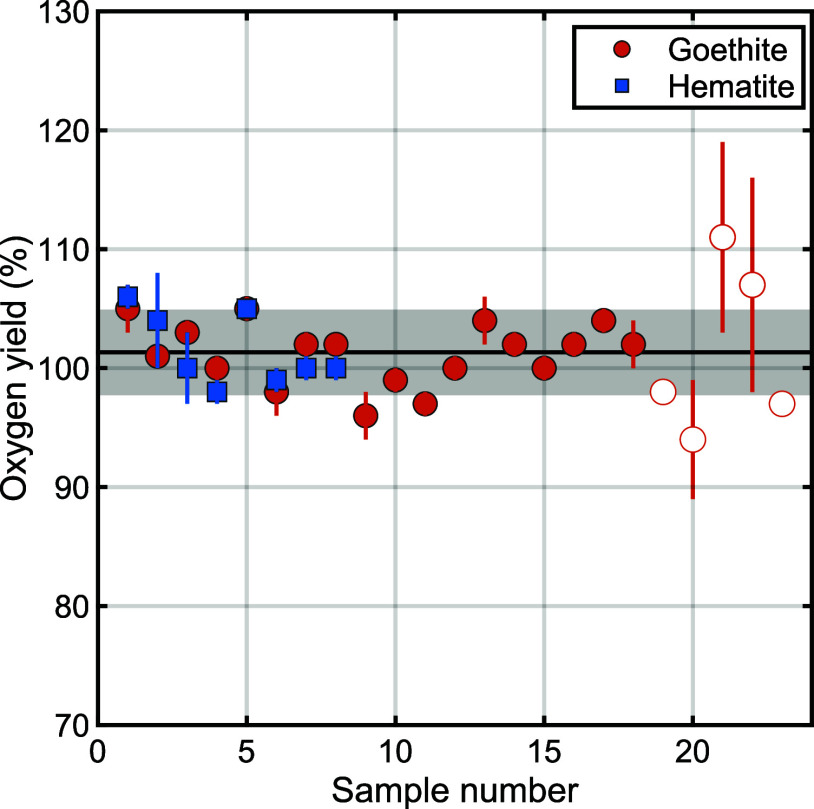
HTC-IRMS oxygen yields for synthetic goethite (orange; orange edges with white centers for samples used for mass-dilution tests) and hematite (blue). Individual markers indicate sample means and vertical bars indicate ±1σ analytical uncertainty for *n* ≥ 3 replicates per sample (Tables S2–S4); gray shaded region is the average ±1σ yield across the entire data set (101.1 ± 3.6%, *n* = 32). Consistent yields of ∼100% across all samples indicate efficient conversion of sample oxygen to CO using the HTC-IRMS method.

Yields for both minerals under all experimental conditions were consistently near 100% (Figure 2, Tables S2–S4). Specifically, yields for any given sample (i.e., average of *n* ≥ 3 analytical replicates) ranged from a minimum mean value of 94% to a maximum of 111%, averaging 101.1 ± 3.6% (μ ± 1σ, *n* = 32) and showed no correlation with mineral type or loaded sample mass (mineral-specific average yields goethite = 100 ± 2%, *n* = 24; hematite = 100 ± 3%, *n* = 8).

### Impact of Sample Mass on δ^18^O

**Figure 3:**
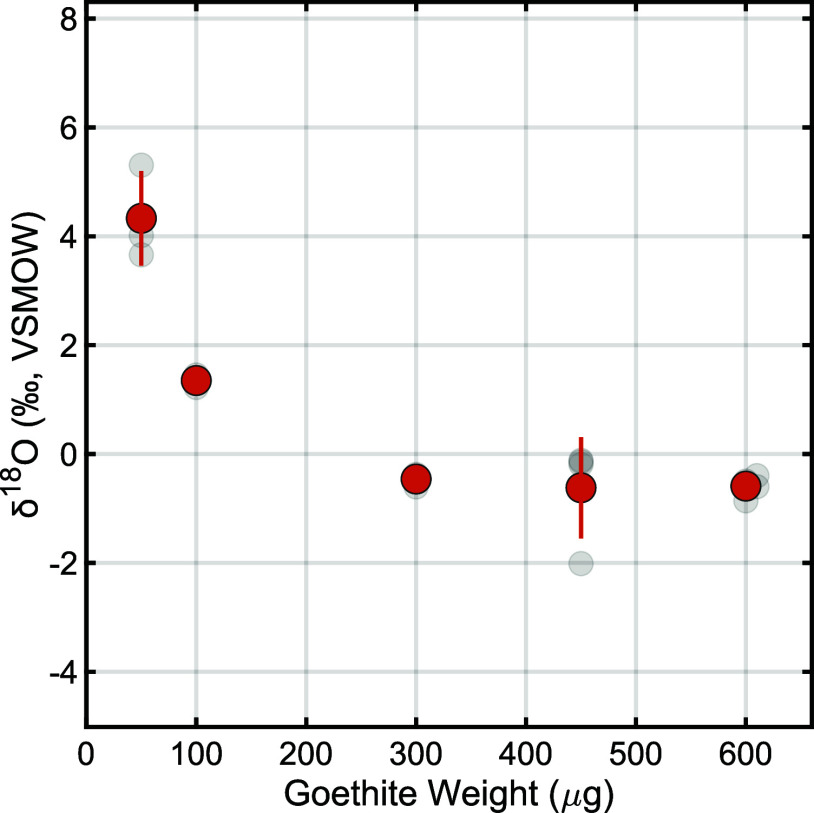
Effect of sample mass on goethite δ^18^O values. Semitransparent gray markers indicate results of individual replicates, orange markers indicate sample means, and vertical bars indicate ±1σ analytical uncertainty for *n* ≥ 3 analytical replicates per sample (Table S2). Sample masses of ≤100 μg show elevated δ^18^O values, likely due to the influence of blank contributions, whereas results stabilize at larger sample masses.

To assess the importance of loaded sample mass on measured isotopic compositions, we performed a dilution series from 50 to 600 μg using a single synthetic goethite sample. Results indicate that small sample masses (i.e., ≤100 μg mineral mass) are shifted by up to ∼5‰ toward heavier δ^18^O values, but that larger sample masses (i.e., ≥300 μg mineral mass) exhibit stable δ^18^O values independent of mass (Figure 3, Table S2). All subsequent results were therefore determined using ≥300 μg sample aliquots. While increasing the sample mass beyond 300 μg further reduces the relative blank contribution, excessively high sample masses may compromise combustion efficiency in the HTC system, representing a trade-off between the sample size and the efficiency of oxygen release.

### Final δ^18^O Values

The resulting goethite δ^18^O values for all synthetic samples using our HTC-IRMS method range from −3.6 ± 0.18‰ to 0.28 ± 0.24‰ (μ ± 1σ analytical error; Table S3). Similarly, hematite δ^18^O values range from 1.03 ± 0.18‰ to 2.78 ± 0.15‰ (Table S4). For both minerals, all samples display an analytical uncertainty of ≤ ± 0.30‰, averaging ±0.15‰ for goethite (*n* = 20) and ±0.13‰ for hematite (*n* = 8).

### Comparison to Laser Fluorination Results

**Figure 4:**
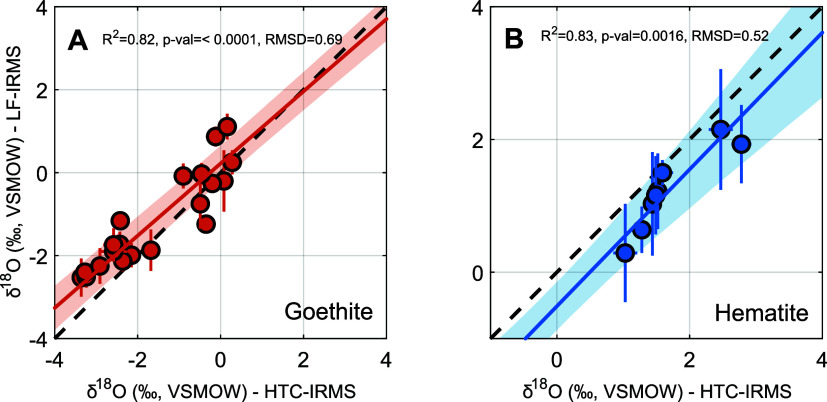
Comparison of δ^18^O values determined by HTC-IRMS (*x* axis) and LF-IRMS (*y* axis) for (A) goethite and (B) hematite. Individual markers indicate sample means whereas horizontal and vertical bars indicate ±1σ analytical uncertainty for *n* ≥ 3 replicates per sample (Tables S3 and S4). Solid orange and blue lines represent the orthogonal-distance regression best-fit lines, and the shaded regions denote the 95% confidence intervals (obtained via 10^4^ Monte Carlo simulations) for these regressions. The root-mean-square deviation (RMSD) from the 1:1 line is also shown. These results demonstrate a strong correlation between HTC-IRMS and LF-IRMS measurements, confirming that HTC-IRMS provides reliable oxygen-isotope data even within the limited isotopic range analyzed.

To assess the validity of our HTC-IRMS method, we compare both goethite and hematite results to those generated previously for the same synthetic samples using LF-IRMS (Tables S3 and S4).^1^ Briefly, fluorination was achieved using a Merchantec 25 W CO_2_ infrared laser spot heat to individual iron oxide samples in the presence of BrF_5_. Following extraction and purification, O_2_ was analyzed on a Thermo Finnigan Delta Plus XL IRMS (details in ref (1)). The resulting δ^18^O values generated by both methods for both minerals are highly correlated about 1:1 (Figure 4; goethite: *R*^2^ = 0.82, *p*-val < 0.0001, RMSD = 0.69‰, *n* = 20; hematite: *R*^2^ = 0.83, *p*-val < 0.005, RMSD = 0.52‰, *n* = 8; where *p*-val indicates statistical significance of orthogonal-distance regression between both methods, and RMSD is the root-mean square deviation from the 1:1 line). Furthermore, with sample masses being comparable, analytical uncertainty for HTC-IRMS results is consistently at least a factor of 2 smaller than that of LF-IRMS for both minerals (goethite average uncertainty: HTC-IRMS = ±0.15‰, LF-IRMS = ±0.31‰; hematite average uncertainty: HTC-IRMS = ±0.13‰, LF-IRMS = ±0.59‰; all uncertainty as ±1σ, *n* ≥ 3 analytical replicates per sample in all cases; Tables S3 and S4). HTC-IRMS achieves high-precision results at sample masses comparable to those used in LF-IRMS, while offering enhanced automation, safety, and reduced handling risks.

## Discussion

### Importance of Sample Mass

Our goethite dilution series reveals that the sample mass can influence measured isotopic compositions, with ≤100 μg aliquots displaying higher, mass-dependent δ^18^O values and ≥300 μg aliquots displaying constant values independent of mass (Figure 3). Consistent yields near 100% for all masses indicate that isotopic deviation at ≤100 μg is unlikely to result from isotopic fractionation or biases toward structural vs hydroxyl oxygen, both of which would be expected only at lower yields. Still, yield uncertainty between analytical replicates is largest for the smallest (i.e., ≤100 μg; yield error ≥ ±5%; *n* = 3) and largest (i.e., 600 μg; yield error ±9%; *n* = 4) sample masses in our dilution series, suggesting some influence of mass on the ability to precisely calculate HTC-IRMS yields (Figure 3; Table S2). Nevertheless, there exists no relationship between the absolute yield or yield uncertainty and the resulting δ^18^O value.

Rather, δ^18^O biases at low sample mass likely result from blank correction inaccuracies and/or instrument nonlinearity for smaller IRMS peak sizes (although the latter is accounted for via linearity corrections during postprocessing). Specifically, smaller sample masses are associated with higher relative blank contributions by peak area: 42.2 ± 1.9% (*n* = 3) for 50 μg aliquots and 28.6 ± 1.2% (*n* = 3) for 100 μg aliquots. In contrast, as the sample mass increases to ≥300 μg, the blank contribution decreases to ≤10% of the total peak area. This results from the fact that the process blank is largely controlled by HTC capturing time and flow rate, which remained constant throughout this study despite varying sample masses (Figure 1). A ≤10% blank area contribution additionally corresponds to the sample mass at which δ^18^O values stabilize (Figure 3), indicating that reliable blank correction can occur when the blank contribution is below this threshold. Thus, to maximize yield precision while minimizing blank corrections, all subsequent analyses were performed with 300–500 μg sample aliquots, thereby enhancing the reliability of the isotopic results.

### Method Comparison and Performance

Comparing our results with those determined by using LF-IRMS for the same set of synthetic minerals provides key insights into the strengths of the HTC-IRMS method. Specifically, δ^18^O values obtained here for both minerals are strongly correlated with those using the LF-IRMS method, demonstrating the reliability of HTC-IRMS as an alternative analytical procedure (Figure 4). The correlation between HTC-IRMS and LF-IRMS measurements was evaluated by using a regression analysis that incorporates an uncertainty envelope (a 95% confidence interval calculated via Monte Carlo simulation). This envelope confirms that the data are consistent with a 1:1 correspondence within expected variability. Still, small discrepancies between methods are observed, likely due to a combination of (i) inherent analytical uncertainties associated with each method, (ii) how the results are calibrated to absolute values (i.e., VSMOW/SLAP2 scale), and (iii) the limited range of δ^18^O values analyzed. Each factor is explored below.

First, HTC-IRMS exhibits improved analytical precision relative to that of LF-IRMS for the sample set analyzed here. Larger uncertainty for LF-IRMS-derived δ^18^O values may arise from several factors, including incomplete lasing, where the fluorination reaction does not fully release oxygen from the sample material.^28^ This issue is further compounded by the manual nature of LF-IRMS, which introduces variability—and thus potential for isotopic fractionation—in handling and cleanup steps. Factors contributing to such variability include laser type, specific irradiation conditions, choice and purity of fluorinating agents (e.g., F_2_ or BrF_5_), and sample size.^29−31^ In contrast, the fully automated HTC-IRMS system minimizes user variability and ensures consistent reaction conditions, contributing to the observed factor of ≥2 improvement in analytical precision observed here.

Second, by measuring both solid and liquid samples on the same analytical system with minimal differences, HTC-IRMS allows for direct calibration to absolute δ^18^O values using internationally accepted water standards (e.g., VSMOW, SLAP2; Figure S2). In contrast, traditional fluorination systems often rely on mineral reference materials such as quartz that have been previously calibrated to water standards (e.g., NBS-28);^32^ such two-step calibration potentially increases uncertainty and decreases accuracy. Nevertheless, the fact that δ^18^O values for both minerals fall around a 1:1 line with no bias when comparing HTC-IRMS vs LF-IRMS measurements—despite independent calibration methods (c.f., ref (1))—strongly suggests that the calibration processes for both instrumental setups considered here are effective and robust (RMSD ≤ 0.69‰; Figure 4).

Finally, the limited range of δ^18^O values in our experiments (∼2 and ∼4‰ for hematite and goethite, respectively) leads to a low signal-to-noise ratio, thus decreasing regression robustness and *R*^2^ values in the comparative analysis (Figure 4). Nevertheless, the observed correlation between HTC-IRMS and LF-IRMS suggests that both methods accurately capture the same isotopic variations despite this limited range.

### Practical Implications and Future Directions

Although this study utilized custom-built hardware, our findings suggest that off-the-shelf elemental analyzer systems—particularly the vario PYRO cube (Elementar; Langenselbold, Germany)—can likely achieve high-precision oxygen-isotope analysis of iron (oxyhydr)oxides with minimal modifications.^33,34^ The most important feature of this design is the “trap and purge” technology. Analogous to our trapping interface, such technology allows for trapping and focusing of CO that is released over ∼1 min during relatively slow reaction kinetics; this is particularly important for minerals such as hematite. In contrast, such slow CO release will lead to poor peak shape in traditional EA systems that utilize packed gas chromatographic columns.

Nevertheless, limitations with off-the-shelf systems do exist. In particular, the vario PYRO cube autosampler is not continuously He-purged, which may increase blanks and decrease precision for hygroscopic minerals such as iron (oxyhydr)oxides and phyllosilicate clays due to water adsorption. Despite these potential drawbacks, the use of such off-the-shelf systems will likely facilitate broader adoption and lower barriers to entry for many laboratories, thus enabling more widespread application of iron (oxyhydr)oxide δ^18^O analysis.

Furthermore, a significant challenge in the field of iron (oxyhydr)oxide oxygen-isotope analysis is the absence of standard materials with a broad range of accepted δ^18^O values (i.e., several tens of permil). The creation and distribution of such standards, measured using multiple methods including HTC-IRMS and LF-IRMS, would provide a robust statistical foundation for intermethod comparisons (cf., Figure 4). Such materials will prove crucial to improve the accuracy and consistency of isotopic data across different laboratories.

## Conclusions

This study establishes the HTC-IRMS method as a robust and practical alternative for high-precision oxygen-isotope analysis of iron (oxyhydr)oxides. Compared with traditional fluorination techniques, HTC-IRMS reduces safety risks and operational complexity while achieving high analytical precision, accuracy, and throughput. Furthermore, the ability of HTC-IRMS to deliver quantitative oxygen yields from submilligram quantities of mineral makes it ideal for high-resolution applications in environmental and geochemical research. The system’s versatility in handling both liquid and solid samples—along with its fully automated processing capability—makes it accessible to a wide range of users, including those without fluorination expertise. This accessibility has the potential to expand the adoption of iron (oxyhydr)oxide isotopic analysis across several laboratories, facilitating broader research efforts.

## Supporting Information Available

Mineral synthesis protocols; calibration procedures and yield determination data; column packing image (Figure S1); system calibration (Figure S2); reference gas calibration data (Table S1); goethite mass-dilution data (Table S2); goethite oxygen-isotope results (Table S3); and hematite oxygen-isotope results (Table S4) (PDF)
