# Supplementary material for: High-Precision Oxygen-Isotope Analysis of Iron (Oxyhydr)oxides Using High-Temperature Conversion Isotope Ratio Mass Spectrometry
Source: Anal Chem. 2025 Mar 28;97(13):7002–9. doi: 10.1021/acs.analchem.4c04676 (PMC11983365; doi:10.1021/acs.analchem.4c04676)
Supplement: Supplementary file 1 — ac4c04676_si_001.pdf [file ac4c04676_si_001.pdf]

Supporting Information for Publication

High-precision oxygen-isotope analysis of iron (oxyhydr)oxides using high-temperature conversion–isotope ratio mass spectrometry

Nir Galili, Anna Somlyay, Giorgia Aquila, Reto Wijker, Philip Gautschi, Lukas Wacker, Jordon D. Hemingway

Geological Institute, Department of Earth Sciences, ETH Zurich, Zurich, 8092, Switzerland

Laboratory of Ion Beam Physics, Department of Physics, ETH Zurich, 8093, Zurich, Switzerland

Supporting Information

Mineral synthesis protocols; calibration procedures and yield determination data; Figure S1 (column packing); Figure S2 (system calibration); Table S1 (reference gas calibration); Table S2 (goethite mass-dilution data); Table S3 (goethite oxygen-isotope results); Table S4 (hematite oxygen-isotope results).

Supporting Information Available

Mineral syntheses

Goethite Goethite was synthesized by dissolving 40 g of $Fe(NO_3)_3 \cdot 9\,H_2O$ in 500 mL of doubly distilled water (DDW, 18.2 $M\Omega\,cm^{-1}$) and adjusting to pH 7 by adding 1 M NaOH. The solution was vigorously stirred, and fresh precipitates underwent a series of rinsing and washing cycles using de-aerated DDW (purged with 99.99 % N_2) followed by a 1-hour N_2-purge to remove any residual O_2 present in the final solution. The deaerated suspension was transferred to an anaerobic glovebox ($O_2 < 1$ ppm), where 10 mL aliquots of ferrihydrite (mineralogically verified by X-ray diffraction; XRD) were transferred into glass bottles. Subsequently, 0.1 M $Fe(NH_4)_2(SO_4)_2 \cdot 6\,H_2O$ was dissolved in a de-aerated 0.1 M HEPES buffer and adjusted to pH 7 using de-aerated 0.5 M NaOH. Five mL of this buffered solution was then introduced into each ferrihydrite-containing glass bottle to achieve a $Fe(II)/Fe_{total}$ ratio of ~ 0.45. Bottles were then vigorously stirred, sealed with butyl rubber stoppers, crimped, and quickly brought to their designated aging temperatures. Experiments were conducted between 4 and 90 °C over time periods of 3 hours to 9 months.

Hematite To synthesize hematite, DDW, 1 M NaOH, and 1 M $NaHCO_3$ solutions were prepared and de-aerated with N_2 gas for 2 hours. Bottles were then sealed and transferred to an anaerobic glovebox, where 3.35 g reagent-grade $FeCl_3 \cdot 6\,H_2O$ was added to 63 mL aliquots of DDW in glass bottles. Subsequently, 8.5 mL of 1 M NaOH was added and intensely stirred

to avoid the formation of aggregates, followed by the addition of 8.5 mL 1 M $NaHCO_3$. All solutions were heated to the designated aging temperature on a hot plate before mixing. Finally, 79 mg $FeCl_2$ was added to all except one control bottle at each temperature to achieve a Fe(II)/Fe_{total} ratio of $\sim$ 0.05. All bottles were then sealed with butyl rubber stoppers, crimped, and vigorously shaken. Experiments were conducted under three aging temperatures—50, 70, and 90 °C—over a time period of 60 days.

Post-synthesis preparation After synthesis, all samples were acid washed with 1 M HCl to remove any residual amorphous phases then washed three times with DDW. Samples were then dried at 60 °C in a desiccator under vacuum. Once dry, samples were homogenized using an agate mortar and pestle then returned to the vacuum dessicator to remain dry until measurements. Dry samples were examined by both XRD and Scanning Electron Microscopy (SEM) to ensure mineralogical purity (*1*). No phase other than goethite or hematite was identified in any sample by either method.

References

(1) Galili, N. et al. The geologic history of seawater oxygen isotopes from marine iron oxides. *Science* **2019**, *365*, 469–473.

(2) International Atomic Energy Agency *Certification report on value assignment for the δ^2H and δ^{18}O stable isotopic composition in the water reference material GRESP (Greenland Summit Precipitation)*; 2021; pp 1–31.

(3) United States Geological Survey *Reference materials and calibration services*; 2023; Accessed: 2023-07-18.

(4) United States Geological Survey *Report of stable isotopic composition reference material W-67400-S water (hydrogen and oxygen isotopes in water)*; 2019; pp 1–3.

(5) International Atomic Energy Agency *Reference sheet for international measurement standards: VSMOW2 Vienna Standard Mean Ocean Water 2, water ($\delta^2 H_{VSMOW\text{-}SLAP}$, $\delta^{18} O_{VSMOW\text{-}SLAP}$); SLAP2 Standard Light Antarctic Precipitation 2, water ($\delta^2 H_{VSMOW\text{-}SLAP}$, $\delta^{18} O_{VSMOW\text{-}SLAP}$)*; 2017; pp 1–8.

(6) International Atomic Energy Agency *Reference sheet for stable isotope reference materials: IAEA-607 (water, low level ^2H and ^{18}O enriched), IAEA-608 (water, medium level ^2H and ^{18}O enriched), and IAEA-609 (water, high level ^2H and ^{18}O enriched)*; 2017; pp 1–8.

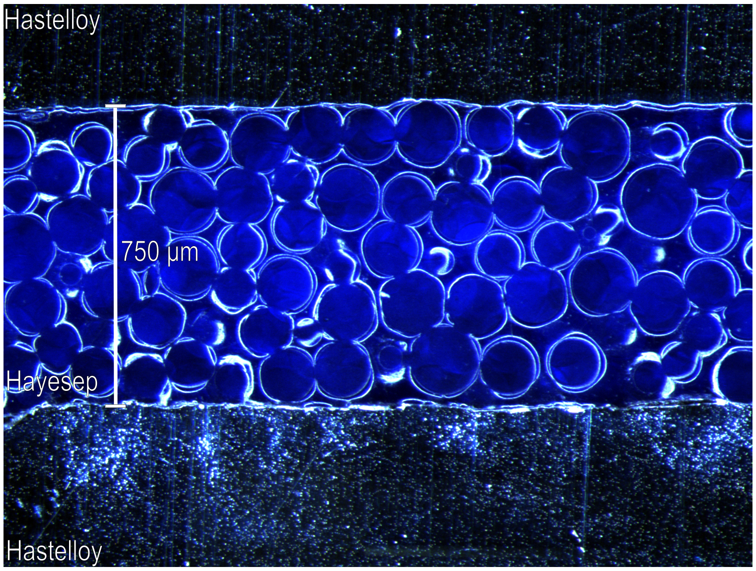

Figure S1: Light microscopy image of packed porous polymer used for T_1 (Hayesep A, 100-120 mesh). A 1 cm section of spare column was embedded into blue stained epoxy resin and polished to 1 µm to achieve a cross-sectional surface for imaging. Based on stained bead diameters (white-to-light blue lines), we estimate a packed porosity of $\phi \sim 11\%$.

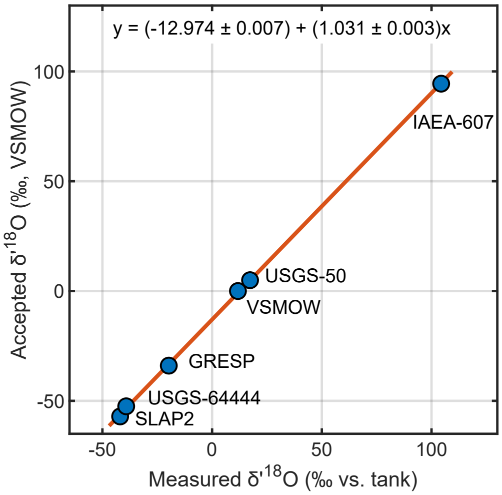

Figure S2: Calibrating the HTC-IRMS system (instrument linearity and reference gas isotopic composition) using internationally distributed liquid reference materials (*2–6*). Blue markers are averages of individual samples (uncertainty smaller than marker symbols); red line indicates orthogonal distance regression best-fit line (regression equation reported with $\pm 1\sigma$ error on regression parameters). Results indicate a tank gas δ'^{18}O value of -12.974 ± 0.007 ‰ VSMOW with a slope of 1.031 ± 0.003, suggesting only minor scale compression (Table S1).

Table S1: HTC-IRMS system calibration results for liquid water standards. Accepted $\delta'^{18}O$ values from Refs. (2–6); all uncertainties are expressed as $\pm 1\sigma$.

Reference material	$\delta'^{18}O$, HTC-IRMS (‰ vs. tank)	n	$\delta'^{18}O$, accepted (‰ VSMOW)
SLAP2	-41.93 ± 0.74	5	-57.10 ± 0.02
USGS - 64444	-39.16 ± 0.55	5	-52.49 ± 0.08
GRESP	-19.75 ± 1.57	4	-33.97 ± 0.04
VSMOW	11.77 ± 0.67	6	0.00 ± 0.30
USGS - 50	17.29 ± 0.67	5	4.94 ± 0.02
IAEA - 607	104.40 ± 0.61	5	94.42 ± 0.13

Table S2: Goethite dilution series δ^{18}O and yield results. All uncertainties are expressed as $\pm 1\sigma$.

Mass (µg)	δ^{18}O (‰ VSMOW)	yield (%)	n
52.50 ± 2.29	4.33 ± 0.87	94 ± 5	3
98.67 ± 1.26	1.35 ± 0.12	111 ± 8	3
299.00 ± 2.86	-0.59 ± 0.20	98 ± 1	4
450.88 ± 3.01	-0.62 ± 0.93	97 ± 1	4
602.13 ± 5.07	-0.46 ± 0.10	107 ± 9	4

Table S3: HTC-IRMS and LF-IRMS oxygen-isotope and yield results for all goethite samples analyzed in this study (yields from HTC-IRMS only). LF-IRMS results from Ref. (1). All uncertainties are expressed as $\pm 1\sigma$.

HTC-IRMS			LF-IRMS	
δ^{18}O (‰ VSMOW)	yield (%)	n	δ^{18}O (‰ VSMOW)	n
-2.15 ± 0.17	105 ± 2	4	-1.99 ± 0.29	4
-0.12 ± 0.20	101 ± 1	4	0.87 ± 0.24	4
-1.68 ± 0.19	103 ± 1	4	-1.87 ± 0.50	4
-2.36 ± 0.08	100 ± 1	4	-2.13 ± 0.25	4
0.08 ± 0.13	105 ± 1	4	-0.20 ± 0.74	4
-0.90 ± 0.21	98 ± 2	3	-0.08 ± 0.30	4
-2.43 ± 0.06	102 ± 1	3	-1.73 ± 0.31	4
-3.36 ± 0.18	102 ± 1	4	-2.53 ± 0.46	4
-3.24 ± 0.08	96 ± 2	4	-2.51 ± 0.26	4
-3.27 ± 0.09	99 ± 1	4	-2.40 ± 0.20	4
-2.42 ± 0.07	97 ± 1	4	-1.16 ± 0.04	4
0.16 ± 0.21	100 ± 1	4	1.11 ± 0.31	4
-0.20 ± 0.09	98 ± 1	4	-0.26 ± 0.05	4
-2.58 ± 0.25	104 ± 2	4	-1.90 ± 0.04	4
-2.91 ± 0.12	102 ± 1	4	-2.25 ± 0.43	4
-2.58 ± 0.25	100 ± 1	4	-1.74 ± 0.40	4
-0.35 ± 0.09	102 ± 1	4	-1.24 ± 0.15	4
0.28 ± 0.24	104 ± 1	4	0.25 ± 0.29	4
-0.49 ± 0.21	102 ± 2	4	-0.75 ± 0.60	3
-0.46 ± 0.10	98 ± 1	4	-0.03 ± 0.26	4

Table S4: HTC-IRMS and LF-IRMS oxygen-isotope and yield results for all hematite samples analyzed in this study (yields from HTC-IRMS only). LF-IRMS results from Ref. (*1*). All uncertainties are expressed as $\pm 1\sigma$.

HTC-IRMS			LF-IRMS	
$\delta^{18}O$ (‰ VSMOW)	yield (%)	n	$\delta^{18}O$ (‰ VSMOW)	n
1.44 ± 0.12	106 ± 1	4	1.03 ± 0.78	3
1.03 ± 0.18	104 ± 4	4	0.29 ± 0.74	3
1.49 ± 0.06	100 ± 3	3	1.16 ± 0.59	3
1.59 ± 0.16	98 ± 1	4	1.50 ± 0.19	3
2.47 ± 0.18	105 ± 1	4	2.15 ± 0.91	3
2.78 ± 0.15	99 ± 1	4	1.93 ± 0.59	3
1.28 ± 0.10	100 ± 1	4	0.64 ± 0.35	3
1.52 ± 0.16	100 ± 1	4	1.22 ± 0.57	3
